# 

**Published:** 1908-09

**Authors:** 


*The following donation has been received since the publication of the List in June:*

*August 31st,* 1908.

R. Shingleton Smith, M.D. (1) .. .. .. 2 volumes.

---

### SIXTY-NINTH LIST OF BOOKS.

The titles of books mentioned in previous lists are not repeated.

The figures in brackets refer to the figures after the names of the donors, and show by whom the volumes were presented. The books to which no such figures are attached have either been bought from the Library Fund or received through the *Journal*.

*Catalogue of Lewis's Circulating Library* .. .. .. New Ed. 1908

**Compton, A. T.** .. *Essentials of Surgery* .. .. .. .. .. .. 1908

*Contributions to Medicine and Surgery by the Faculty of the New York Post-Graduate School* .. .. .. .. .. .. .. .. 1908

*Encyclopedia and Dictionary of Medicine and Surgery* Vol. VIII. 1908

**Herbert, H.** .. .. *Cataract Extraction* .. .. .. .. .. .. 1908

**Hewer, Mrs. J. L.** *Our Baby* .. .. .. .. .. .. 11th Ed. 1908

**Hutchison and H. Rainey, R.** *Clinical Methods* .. 4th Ed. 1908

**Leduc, S.** .. .. *Electric Ions and their use in Medicine.* (Tr. by R. W. Mackenna) .. .. .. .. 1908

**Litchfield, W. F.** .. *Diphtheria in Practice* .. .. .. .. .. .. 1908

**Newman, B. A. Whitelegge and G.** *Hygiene and Public Health* New Ed. 1908

**Osler, W.** .. .. *Aequanimitas* .. .. .. .. .. .. (1) 1905

**Parsons, J. H.** .. *The Pathology of the Eye.* Vol. IV., Pt. 2.. 1908

**Rainey, R. Hutchison and H.** *Clinical Methods* .. 4th Ed. 1908

**Sawyer, J.** .. .. *Points of Practice in Maladies of the Heart* (1) 1908

**Thomson, J.** .. *Clinical Examination and Treatment of Sick Children* .. .. .. 2nd Ed. 1908

**Walsh, J. F.** .. *History of the Medical Society of the State of New York* .. .. .. .. .. .. .. .. 1907

**Whitelegge and G. Newman, B. A.** *Hygiene and Public Health* New Ed. 1908

### TRANSACTIONS, REPORTS, JOURNALS, &c.

| | | |
|---|---|---|
| American Journal of the Medical Sciences, The | Vol. CXXXV. | 1908 |
| American Pediatric Society, Transactions of the .. | Vol. XIX. | 1908 |
| Archives de Neurologie .. .. .. .. | 3me ser., Tom. I., II. | 1907 |
| British Medical Journal, The .. .. .. .. .. | Vol. I. for | 1908 |
| Clinical Journal, The .. .. .. .. .. .. .. .. | Vol. XXXI. | 1908 |
| Edinburgh Medical Journal, The .. .. .. | N.S., Vol. XXIII. | 1908 |
| Glasgow Medical Journal, The .. .. .. .. .. | Vol. LXIX. | 1908 |
| Hospitals—St. Thomas's Hospital Reports .. .. | Vol. XXXV. | 1908 |
| Lancet, The .. .. .. .. .. .. .. .. .. .. | Vol. I. for | 1908 |
| Medical Record .. .. .. .. .. .. .. .. | Vol. LXXIII. | 1908 |
| Medical Society of London, Transactions of the .. | Vol. XXIX. | 1906 |
| Münchener medizinische Wochenschrift .. .. .. | Bd. I. für | 1908 |
| Nordiskt medicinskt Arkiv .. .. .. .. .. .. .. .. | | 1906 |
| Practitioner, The .. .. .. .. .. .. .. .. | Vol. [LXXX.] | 1908 |
| Progressive Medicine .. .. .. .. .. .. .. .. | Vol. II. | 1908 |
| Royal Academy of Medicine in Ireland, Transactions of the | Vol. XXVI. | 1908 |
| Scottish Medical and Surgical Journal, The .. .. | Vol. XXII. | 1908 |
| Tuberculosis, Royal Commission on, Reports .. .. | 7 vols. | 1904—1907 |

### Local Medical Notes.

---

**University College, Bristol.**—In examinations recently held :—

M.D. Lond.—*Midwifery and Diseases of Women:* W. J. H. Pinniger.

Inter. M.B. Lond.—H. B. Parker, W. W. Pratt.

Conjoint Board, London.—*Practical Pharmacy:* G. F. Fawn, F. C. Nicholls. *Anatomy and Physiology:* I. Hudleston, S. Marle, V. Pinnock. *Medicine:* *C. Clarke, E. E. Davies, B. Stone. *Surgery:* P. J. Martin, *F. T. Boucher. *Midwifery:* F. C. Morgan, T. B. Dixon.

We have been asked to state that owing to an oversight in the recently issued Prospectus of University College, the name of Dr. Barclay J. Baron, Consulting Physician to the Throat and Nose Department at the Bristol General Hospital, was omitted from the list of the Hospital Staff.

**Royal Infirmary.**—J. R. Charles, M.D., B.C. Cantab., and J. A. Nixon, M.B., B.C. Cantab., have been appointed Honorary Physicians.

J. M. Fortescue-Brickdale, M.D., B.Ch. Oxon., has been appointed Honorary Assistant Physician.

At the half-yearly meeting of the governors of this institution the medical report stated that during the past six months 1,747 in-patients were admitted and 20,227 out-patients were treated. The daily average number of in-patients was 235. The financial statement showed that the income was £8,420, and expenditure amounted to £8,416. The total adverse balance of the Infirmary is now £3,326.

**Medical Inspectors of Schools.**—The following have been appointed by the Bristol Education Committee :—T. M. Carter, M.D. Durh., M.R.C.S., L.R.C.P. Lond. ; T. A. Green, M.D., C.M. Edin. ; W. T. Maddison, M.D. Lond., M.R.C.S. Eng. ; E. Cecil Williams, M.B., B.C. Cantab. ; Annie F. M. Cornall, F.R.C.S. Irel., L.R.C.P. Edin.

**Hospital Sunday Fund in Bristol.**—The total amount contributed to this fund in Bristol for 1908 was £1,859, being £104 in excess of the 1907 collection. £1,776 have been divided amongst the various medical charities of the city.

* Denotes complete examination.

### PUBLICATIONS RECEIVED.

From Baillière, Tindall & Cox, London:

Cataract Extraction. By H. Herbert, F.R.C.S. 1908. Price, 12s. 6d. net.

From Cassell & Company, Limited, London:

*Clinical Methods.* By Robert Hutchison, M.D., and Harry Rainey, M.D. Fourth Edition. 1908. Price, 10s. 6d.

*Hygiene and Public Health.* By B. Arthur Whitelegge, C.B., M.D., and George Newman, M.D. New and Revised Edition. 1908. Price, 7s. 6d.

From J. & A. Churchill, London:

*Transactions of the Ophthalmological Society of the United Kingdom.* Vol. XXVIII., Fasc. II. 1908. Price, 4s. per part.

From Henry Frowde and Hodder & Stroughton, London:

*The Pathology of the Eye.* By J. Herbert Parsons, B.S., D.Sc. Volume IV.—Part II. 1908. Price, 15s. net.

From William Green & Sons, Edinburgh:

*Green's Encyclopedia and Dictionary of Medicine and Surgery.* Vol. VIII.—*Physiology (Nutrition)—Rhinolalia.* [1908.]

*Clinical Examination and Treatment of Sick Children.* By John Thomson, M.D. Second Edition. 1908. Price, 12s. 6d. net.

From Henry Kimpton, London:

*Essentials of Surgery.* By Alwyne T. Compton, F.R.C.S. 1908. Price, 4s. net.

From H. K. Lewis, London:

*Catalogue of Lewis's Medical and Scientific Circulating Library.* New Edition. 1908.

From Rebman Limited, London:

*Electric Ions and their Use in Medicine.* By Professor Stephane Leduc. Translated by R. W. Mackenna, M.D. 1908.

From Williams & Norgate, London:

*Internationales Centralblatt für die gesamte Tuberkulose-Forschung.* II. *Jahrgang.* Nr. 9. 1908.

From John Wright & Sons, Ltd., Bristol:

*Our Baby: for Mothers and Nurses.* By Mrs. J. Langton Hewer. Eleventh Edition. 1908. Price, 1s. 6d.

